# SCENE: Signature Collection for Endometrial Cancer Prognosis

**DOI:** 10.1111/jcmm.70762

**Published:** 2025-08-03

**Authors:** Erica Dugo, Francesco Piva, Matteo Giulietti, Luca Giannella, Andrea Ciavattini

**Affiliations:** ^1^ Department of Specialistic Clinical and Odontostomatological Sciences Polytechnic University of Marche Ancona Italy; ^2^ Woman's Health Sciences Department, Gynecologic Section Polytechnic University of Marche Ancona Italy

**Keywords:** database, endometrial cancer, gene expression profiling, prognostic biomarkers, survival prediction, transcriptomic signatures

## Abstract

Endometrial cancer (EC) is the most common malignancy of the female reproductive tract; its prognosis is difficult to predict. Despite the technique of single‐cell transcriptomic analysis (scRNA‐seq) returning single‐cell level expression data and promising to improve the accuracy of prognosis prediction, a tool that correlates transcriptomic signatures with survival is missing. To this aim, we have created SCENE, a database that collects information to correlate EC transcriptomic signatures with patient prognosis. We performed a review of the literature present in PubMed to collect transcriptomic signatures annotated with their characteristics, differential expression between healthy and sick patients, between patients with more and less favourable prognosis, and cellular pathways in which the genes are involved, as well as references to the original studies. The analysis of about 200 studies has allowed us to obtain 700 mRNA signatures, 60 microRNA (miRNA), and 150 long non‐coding RNA (lncRNA), involved in 60 molecular pathways. Each signature is annotated with its specific prognostic outcome that it influences, such as overall survival (OS), progression‐free survival (PFS), relapse‐free survival (RFS), and disease‐specific survival (DSS). The SCENE resource collects and annotates information that is widespread in the literature to facilitate the interpretation of transcriptomic data obtained with any technique in EC. In the case of scRNA‐seq data, SCENE may reveal cells predisposed to develop therapy resistance and metastasis.

**Abbreviations**
CNH: copy‐number high
CNL: copy‐number low
CNVs: copy number variations
DSS: disease‐specific survival
EC: endometrial cancer
EMT: epithelial‐mesenchymal transition
FFPE: formalin‐fixed and paraffin‐embedded
FIGO: International Federation of Gynecology and Obstetrics
H&E: haematoxylin and eosin
IHC: immunohistochemistry
lncRNA: long non‐coding RNA
miRNA: microRNA
MMRd EC: DNA mismatch repair‐deficient endometrial cancer
mRNA: messenger RNA
NIRS: CD8+ T‐cell immune risk score
NSMP EC: no specific molecular profile endometrial cancer
OS: overall survival
p53abn EC: p53 abnormal endometrial cancer
PFS: progression‐free survival
POLEmut EC: POLE ultramutated endometrial cancer
RFS: relapse‐free survival
RT‐qPCR: quantitative reverse transcription PCR
scRNA‐seq: single cell RNA sequencing
TCGA: The Cancer Genome Atlas
Tfh: follicular helper‐like T‐cell

## 1 Introduction

Endometrial cancer (EC) is the most common malignancy of the female reproductive tract, with incidence rates rising globally. The American Cancer Society estimates that approximately 69,000 new cases will be diagnosed in the United States in 2025, with this number expected to grow further due to lifestyle and demographic factors [1]. Most cases of EC are diagnosed at an early stage, which allows for early diagnosis and better survival outcomes [2]. However, for patients with advanced or metastatic disease, the prognosis remains poor, with metastases commonly spreading through the lymphatic system to pelvic and para‐aortic lymph nodes, or via hematogenous routes to distant organs such as the lungs, liver, and bones [3]. In particular, several studies have indicated that lymph node invasion is an important prognostic factor with a higher risk of recurrence and poorer survival rates [4]. However, it is not known precisely how this event is related to prognosis, as it is not always correlated with the aggressiveness of cancer. Moreover, other factors also influence prognosis: age, obesity, sociodemographic conditions, FIGO tumour stage, grade, histological subtype, and molecular characteristics [5, 6]. The International Federation of Gynecology and Obstetrics (FIGO) classifies EC according to its extent and helps guide treatment decisions for patients with this tumour. FIGO staging defines that stage I EC is confined to the endometrium or myometrium; in stage II, the tumour extends to the cervix; in stage III, patients have local and/or regional spread with metastases to pelvic and/or para‐aortic lymph nodes. Lastly, in stage IV, the tumour has distant metastases including intra‐abdominal and/or inguinal lymph nodes [7]. Early‐stage disease (Stage I and II) generally has a favourable prognosis, with a 5‐year survival rate exceeding 80% [8]. Advanced‐stage disease (Stage III and IV) and high‐grade histology, such as serous and clear cell carcinomas, are associated with a poorer prognosis.

Regarding molecular characteristics, The Cancer Genome Atlas Research Network (2013) [9] identified four molecular subtypes of endometrial cancer: POLE ultramutated (POLEmut EC), MSI hypermutated (MMRd EC), copy‐number low (NSMP EC), and copy‐number high or p53abn EC, each with distinct clinical outcomes. For example, patients with the POLEmut EC mutational profile have a favourable prognosis, the high copy‐number subtype, often associated with p53 mutations, has a poor prognosis, and MMRd EC and NSMP EC patients have an intermediate prognosis.

However, to better stratify patients and guide treatment decisions, there is an urgent need to enhance our prognostic capabilities both by trying to better understand how the aforementioned indicators interact and by exploring new indicators [10]. In this way, one important area of research is investigating the relationship between cancer gene transcriptional signatures and prognosis. Although these gene expressions are not yet adopted in the clinic, they are providing specific information at the level of tumour cell behaviour. For example, they could detect early epithelial‐mesenchymal transition (EMT), an increase in the proportion of cancer stem cells, the establishment of immune evasion, or angiogenesis.

One of the most promising investigation tools in cancer research is the single‐cell transcriptomic analysis (scRNA‐seq), which is revolutionising the study of gene expression by enabling the analysis of individual cells within a heterogeneous tissue, providing an unprecedented level of resolution for identifying molecular signatures [11]. This technique has such high sensitivity that it is now possible to detect even a few tumour cells carrying specific signatures. The detection of a subpopulation of cells is important in facilitating more accurate prognostic predictions because subpopulations might develop as a new clone that will drive the tumour behaviour [12].

In recent years, several databases collecting biomarkers, such as single genes and transcriptomic signatures, have been developed [13, 14]. However, there is no database specifically dedicated only to EC, and currently, among general cancer databases, none shows a correlation between transcriptomic signatures and the prognosis of EC patients. Among the pan‐cancer databases, we can find, for example, the MarkerDB 2.0 database [15] which collects and provides information on biomarkers specific to various diseases, but in the section on ‘prognostic markers’, there is no reference to EC, and in the section on ‘risk markers’, there are no correlations with EC‐associated genes. Another resource is TheMarker database [16], which constitutes an exclusive database for therapeutic biomarkers, including data on genetic, proteomic, and single‐cell transcriptomic markers, useful for the diagnosis and prognosis of various diseases, with a particular focus on cancers after pharmacological treatment. Regarding EC, the database mainly includes information on treatments with carboplatin, Dostarlimab, and Lenvatinib, but it does not contain biomarkers related to the prognosis of EC patients. DiSignAtlas [17] is another database that collects and analyses transcriptomic signatures to provide information on gene expression patterns that can be used to predict the course of diseases like cancer. However, when searching for information on EC, genes differentially expressed between healthy and diseased patients (i.e., possible diagnostic biomarkers) are returned, but without information on which of these genes show a correlation with prognosis. Gene signatures obtained from these databases can be used by some software to analyse the data generated by scRNA‐seq and reveal the cells carrying these signatures. Among these tools, we cite UCell, which for each signature found in a cell returns a gene signature score that estimates similarity between the searched pattern and the one found [18].

In this study, we developed the SCENE (Signature Collection for Endometrial Cancer Prognosis) database that collects genes whose expressions are associated with patient prognosis, in relation to overall survival (OS) and/or progression‐free survival (PFS), relapse‐free survival (RFS) and disease‐specific survival (DSS). The main use of our database is to exploit well‐known expression data from literature in order to better understand data obtained from scRNA‐seq experiments. This will in turn make it easier to establish a link between RNA data and the prognosis of EC patients.

## 2 Materials and Methods

We performed a literature search in PubMed database using the following keywords: ‘endometrial cancer and gene signature’ and ‘endometrial cancer and gene expression,’ and included studies published between 2008 and 2025. Subsequently, only studies fulfilling the following criteria were selected: (i) those reporting transcriptomic signatures (via high‐throughput technologies) obtained from biopsy tissues of human ECs, or from analysis of TCGA‐EC data, (ii) among the studies remaining after previous filtering steps, we retained those reporting messenger RNA (mRNA), long non‐coding RNA (lncRNA) and microRNA (miRNA), (iii) among the studies that survived the previous filtering stages, we selected those showing transcripts correlated with prognosis with a significance *p*‐value < of 0.05. Reviews, studies with < 20 patients, studies involving animals, xenografts, cell lines, and RNA from blood or swab samples were excluded. The database that collects the signature or single gene expression data obtained from these studies was organised with the following fields:

- *Gene*: official name of the gene related to prognosis according to HGNC (HUGO Gene Nomenclature Committee) and miRBase.
- *Gene ID*: unique identifiers corresponding to NCBI Gene database.
- *Ensembl ID*: unique identifiers corresponding to Ensembl database.
- *RNA*: this indicates if the transcript is mRNA, miRNA, or lncRNA.
- *Description*: the full gene name approved by the HGNC.
- *Expression (among patients)*: differential expression levels (up or down) between two groups of patients with different prognosis (e.g., better versus worse prognosis).
- *Expression levels between healthy subjects and patients*: here it is indicated whether the transcript is over or under expressed in EC patients compared to healthy subjects.
- *OS, PFS, RFS, and DSS Status*: phenotype with which the expression of the gene annotated in the current record has been correlated. This section details the overall survival (OS), progression‐free survival (PFS), recurrence‐free survival (RFS), and disease‐specific survival (DSS) associated with the gene.
- *Pathways/Conditions*: this field lists the biological pathways, cellular functions, or patient conditions in which the gene is involved.
- *Notes*: this section includes additional information such as the association of the gene signature with the FIGO stage, molecular classification, histological grade, histological type, and the presence or absence of metastases, if specified in the study.
- *PMID*: the unique article code for direct access in PubMed.
- *Reference*: provides the bibliographic information needed to identify and locate the scientific article, not only in PubMed but also in other databases.

The endometrial cancer database was developed using standard web technologies, including HTML, CSS, and JavaScript. The database is presented through an interactive web platform that allows users to view, filter, and sort the data dynamically in a table format. Each table column is labelled according to the relevant headers, including Gene, RNA, Description, Expression (among patients), OS, PFS, RFS, DSS, Expression (EC vs. healthy), Pathways/Conditions, Note, PMID, and Reference. The interactivity of the platform was powered by JavaScript, which enables the dynamic sorting, filtering, paginating of data, and data export.

## 3 Results

The literature search returned a total of 3392 unique studies and, after selecting only those reporting transcriptomic signatures obtained from human EC biopsy tissues, we identified 188 studies. Among them, only 11 specifically examined the prognosis in women treated with chemotherapy or radiotherapy. A total of 156 articles utilised RNA sequencing data from TCGA as their primary sample source. A total of 6 articles assessed tumour purity percentages, which reflected the ratio of tumour cells to stromal cells in biopsy samples. In the majority of these studies, the tumour purity was greater than 70% based on haematoxylin and eosin (H&E) staining of histological sections assessed by gynaecologic pathologists. 77 articles included clinical‐pathological characteristics of EC patients, and the range of the number of patients examined in the recovered studies ranged from as few as 28 to as many as 1645. Most of the studies were conducted in China, with 164 articles from this country, followed by Norway and the United States, both with 9 studies.

In terms of experimental techniques, RNA‐sequencing (RNA‐seq) was predominantly used, followed by microarray and quantitative Reverse Transcription PCR (qRT‐PCR). Validation of the expression data was primarily performed using qRT‐PCR and immunohistochemistry (IHC). Approximately 62% of studies associate the detected transcriptomic signature with the age and clinico‐pathological characteristics of EC patients, specifically the FIGO stage, histological grade, and histological type. Fewer studies have examined correlations with molecular subtypes and other factors, such as the presence or absence of metastases.

The database includes 147 articles focusing on mRNAs, 36 articles on lncRNAs, and 14 articles on miRNAs. According to the selected studies, we extracted a total of 1117 single and multiple gene signatures related to prognosis in EC, and they were used to build the SCENE database. The 1117 signatures collectively comprise a total of 913 unique genes (696 mRNAs, 154 lncRNAs and 63 miRNAs) whose expressions were associated with prognosis and, generally, have an impact on the OS of EC patients from the second year of disease. Each of the 1117 database records was annotated with detailed information such as: gene symbol, full name, NCBI gene ID, Ensembl ID, RNA type (i.e., mRNA, lncRNA or miRNA), expression levels of each gene among patients with different prognosis (e.g., up or down), expression differences between patients and healthy subjects, the associated biological pathways or clinical conditions, a “Notes” section with additional insights from the literature review, as well as bibliographic references. In addition, clinical outcomes are detailed through fields such as OS, PFS, RFS, and DSS. Among these, OS is the most frequently reported outcome, appearing alone in 942 (84%) signatures, whereas DSS is the least represented, being reported alone in only 36 (3%) cases. The SCENE database also makes it easy to identify genes whose expression is associated with multiple clinical outcomes. For example, 58 (5%) signatures correlate with both OS and PFS, 29 (3%) signatures are simultaneously associated with OS and DSS, and 39 (4%) signatures are related to both OS and RFS. Overall, 868 (78%) signatures have been associated with poor prognosis, whereas 249 (22%) signatures are correlated to a favourable prognosis.

Our database contains genes that relate to 55 cellular pathways associated with prognosis (Table 1). The more extensively studied transcriptomic signatures were related to cell death, both programmed and non‐programmed, and include genes involved in apoptosis, necrosis, ferroptosis, cuproptosis, and other forms of regulated cell death. The immune cell‐related transcriptomic signatures, shown in Table 1, include genes associated with the CD8+ T‐cell immune risk score (NIRS) and a follicular helper‐like T‐cell (Tfh) infiltration risk model (TIRM). SCENE also contains epigenetic regulators, such as EZH2 and BMI1, which may abnormally influence gene transcription in the EC [18]. In addition, there are angiogenesis‐related markers, including ANGPT1, CD40LG, HIF1A, CCL5, and GPR87, which could contribute to tumour vascularisation [19].

**TABLE 1:** The 55 unique pathways referred to transcriptomic signatures collected in SCENE are shown.

Transcriptomic signatures related to	Number of studies	Transcriptomic signatures related to	Number of studies
Apoptosis	1	Immune cell	3
Autophagy‐dependent cell death (ADCD)	10	Immune checkpoint related genes (ICG)	1
Autophagy‐dependent cell death (ADCD) and apoptosis	1	Immune microenvironment	1
Calcium	1	Immunogenic cell death (ICD)	3
Cell cycle pathway	1	Inflammatory response	3
Cholesterol homeostasis	1	Interferon regulatory factor (IRF)	1
Cuproptosis	4	Kinase	1
Disulfidopsis	1	Lactate metabolism	2
Disulfidptosis/ferroptosis	1	Lymph node metastasis	2
Endoplasmic reticulum (ER) stress	1	Metabolism	3
Epithelial‐mesenchymal transition (EMT)	6	Mitochondrial	1
Oestrogen/progesterone related gene	3	NAD+ metabolism	1
Fatty acid metabolism (FAM)	2	Oxytocin	1
Ferroptosis	6	Prognostic genes related to serous carcinoma of the uterus (USC)	1
Focal adhesion	1	Programmed cell death (PCD)	1
G protein‐coupled receptors (GPRs)	1	Pyroptosis	4
Gelsolin superfamily	1	Redox	1
Genomic instability (GI)	2	Related to the stemness index (mRNAsi)	1
Glucose metabolism	1	Sialylation	1
Glutamine metabolism	1	Sonic Hedgehog (SHH) pathway	1
Glycerophospholipid	1	Stemness	1
Glycolysis	4	TP53 Pathway	1
Glycometabolism and lipid metabolism	1	Tumour microenvironment (TME)	1
Glycosylation	1	Tumour necrosis	6
Hypoxia/Angiogenesis	8	Tumourigenesis and prognosis	76
IFN‐γ	1	Ubiquitination	1
Immune system	11	Vascular invasion	2
Epigenetic regulators	2		

The mRNA most frequently associated with prognosis is CDKN2A, present in 13 articles, followed by NR3C1, present in 6 studies, TPX2 and TNF both present in 5 studies. In particular, CDKN2A overexpression correlates with shorter OS and PFS, advanced FIGO stage, high histological grade, age over 60 years, CNH molecular subtype, and mixed‐serous histology [19, 20, 21]. Similarly, NR3C1 overexpression is associated with a shorter OS, advanced FIGO stage, age over 65 years, mixed‐serous histology, and high histological grade [22, 23]. Overexpression of TPX2 is also indicative of a poor prognosis with shorter OS, FIGO stages III–IV, age over 60 years, histological grade G3, and mixed‐serous histology. Furthermore, patients with endometrial endometrioid carcinoma (EEC) and high expression of TPX2 survived less than 5 years after diagnosis compared to the long‐term survivors [24]. In these studies, the overexpression of TNF is consistently associated with poor clinical outcomes, including shorter OS, PFS, DSS, and RFS [25, 26]. The overexpression of this gene is also associated with histological grade G3, age over 55 years, and serous histology [22, 23].

Among the collected mRNAs, some encode transcription factors, such as HOXB9, NR3C1, STAT1, GATA4, FOXP2, KLF8, and CREB3L3. Interestingly, high CREB3L3 expression levels have been associated with a worse prognosis in EC patients and a more effective response to therapies such as AKT inhibitors, Bicalutamide, Docetaxel, and Temsirolimus, while showing less efficacy with Pyrimethamine and Rucaparib [27]. Furthermore, our database contains receptors including ESR1, FGFR2, and VDR, which are recognised for their roles in cellular responses and tumour development [27, 28]. For example, one study showed that vitamin D nuclear receptor (VDR) expression is significantly higher in endometrial tumour samples with a lower histological grade [28].

Among the long non‐coding RNAs (lncRNAs) collected in our resource, the most frequently identified is BX322234.1, reported in four studies [29, 30, 31, 32]. In addition, AL035530.2, RAB11B‐AS1, SCARNA9, SOS1‐IT1, and AC019080.5 are each mentioned in three articles [32, 33, 34, 35, 36, 37, 38, 39, 40, 41]. Upregulation of the lncRNAs AL035530.2, SOS1‐IT1, AC019080.5, and BX322234.1 in EC patients has been associated with shorter OS. These lncRNAs are related to hypoxia, pyroptosis, cuproptosis, tumour necrosis, and genomic instability (GI). Specifically, the expression level of AL035530.2 in EC correlates with advanced FIGO stage and high histological grade; and upregulation of BX322234.1 and SOS1‐IT1 is associated with FIGO stage III –IV, histological grade G3, age over 60 years, and mixed‐serous histology. Regarding the lncRNA RAB11B‐AS1, one study showed that its upregulation is associated with a favourable prognosis, including longer OS, FIGO stage I–II, histological grade G1, and negative lymph node status [36]. In contrast, two other studies report that upregulation of RAB11B‐AS1 is associated with shorter OS, particularly with FIGO stage III–IV, histological grade G3, age over 60 years, and mixed‐serous histology [32, 33]. Lastly, upregulation of SCARNA9 in EC patients was associated with a favourable prognosis, including longer OS, particularly in those with histological grade G1 [37, 38, 39].

The miRNA most frequently found to be associated with EC prognosis is hsa‐miR‐7‐5p, which appears in three separate studies, according to which its upregulation is linked to a shorter disease duration [42, 43, 44]. Other miRNAs identified by multiple studies include miR‐34b‐5p, miR‐34c‐3p, and miR‐34c‐5p, whose downregulation is associated with poor prognosis and the presence of lymph node metastasis [45, 46].

However, we also encountered contradictory findings relating to certain genes in the literature. For example, overexpression of the GMPPB gene was associated with favourable prognosis in one study [47] and with unfavourable prognosis in another study [48]. Similarly, a study of inflammatory response‐related genes reported that downregulation of the MYC gene is associated with a poor prognosis [49], while another study reported that its overexpression is associated with a shorter OS [50].

**FIGURE 1:**
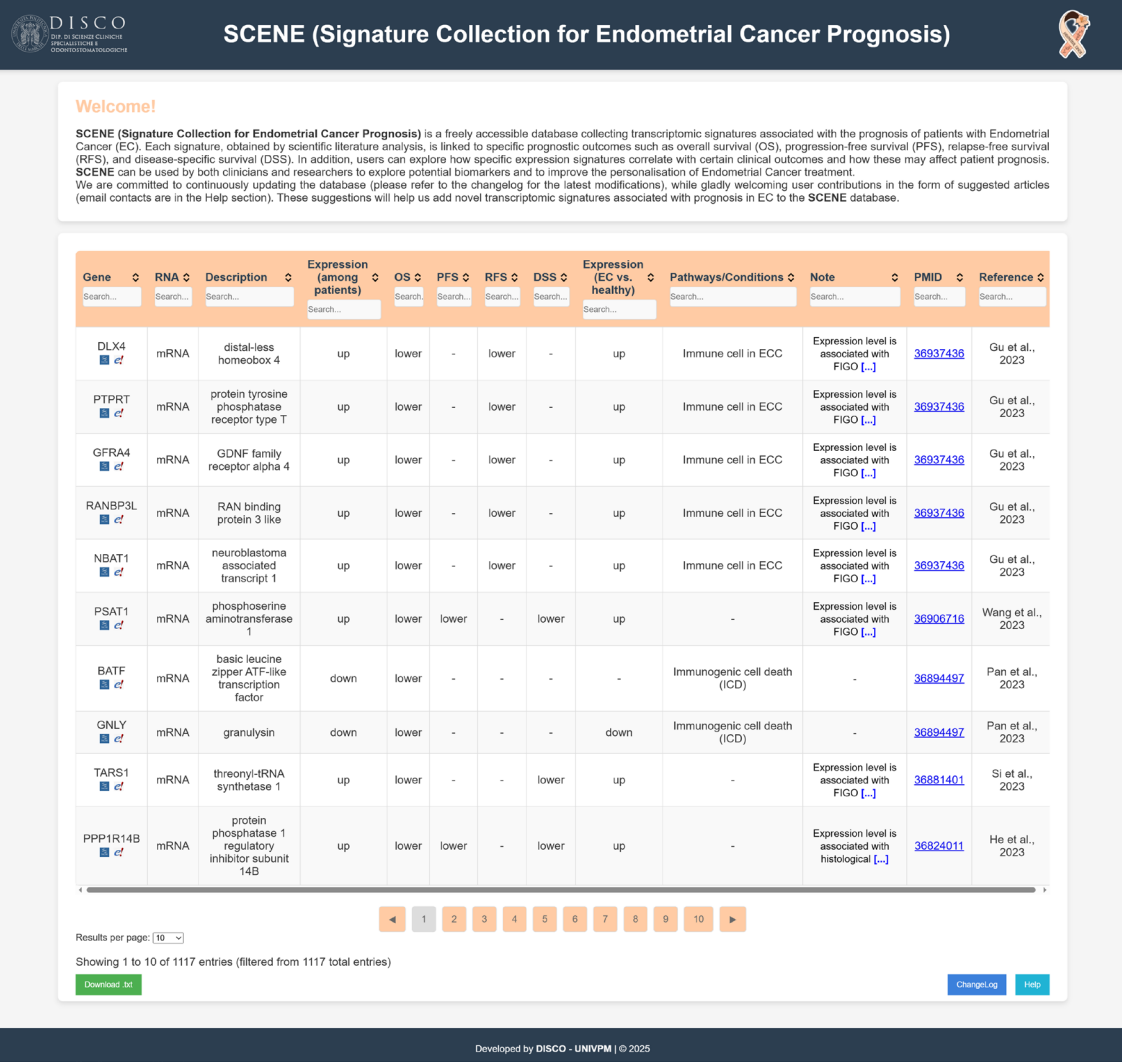
SCENE web interface. Key data fields include Gene, GeneID, and EnsemblID, which provide insights into the specific genes and their associated identifiers. The column “Expression (among patients)” reflects gene expression patterns (up or down) observed in the patient groups (with better or worse prognosis), giving an understanding of how gene expression correlates with disease progression. In particular, clinical outcomes are captured through columns such as OS, PFS, RFS, and DSS. In addition, the differential gene expression levels in EC patients versus healthy subjects (column “Expression (EC vs. healthy)”) are collected. Other columns such as Pathways/Conditions describe the pathway to which the gene belongs or the clinical condition determined by the gene; Note column provides additional observations on the data obtained by literature analysis.

The SCENE database serves as a comprehensive, expertly curated platform that brings together all rigorously validated transcriptomic biomarkers with confirmed prognostic and predictive relevance in EC. By consolidating this information into a single resource, SCENE streamlines access to critical molecular data, supporting both scientific research and the development of more personalised therapeutic strategies. In order to make our results easily accessible to researchers and clinicians, we created an interactive web platform (http://www.introni.it/EC/SCENE.html) (Figure 1) that allows users to view the data in a paginated table format, sort, and filter the database records. For instance, SCENE allows filtering of transcriptomic signatures that are associated with various clinico‐pathological parameters, such as histological grade, FIGO stage, or molecular subtype of EC. Similarly, the user can select a gene and filter the signatures associated with a specific clinical outcome, such as reduced OS. In addition, users can select signatures associated with particular RNA types, for example, only signatures associated with lncRNA. Alternatively, users can apply combined filters to obtain more specific results. For example, it is possible to select signatures associated with several clinical outcomes simultaneously, such as OS and PFS. Other examples are signatures consisting exclusively of miRNAs with favourable prognosis, or signatures including only lncRNAs identified in ECs with histological grade G3 and associated with poor prognosis. Finally, users can download the entire dataset, or just the filtered data, in text format for further analysis. For example, some transcriptomic scoring tools (such as AUCell, UCell, JASMINE, etc.) for scRNA‐seq data exist, which are useful for the identification of subpopulations within a tumour that exhibit high‐risk molecular profiles. If SCENE signatures were utilised by these scoring tools, it would improve patient stratification according to prognosis, ultimately enhancing clinical decision‐making.

## 4 Discussion

In this study, a literature review was conducted to collect the gene expressions associated with the prognosis of endometrial carcinoma patients, together with their related information on prognosis and the cellular pathways involved. With this data, a web database was developed to make these prognostic signatures accessible to researchers and clinicians, facilitating both scientific research and the advancement of more personalised treatment approaches, for example, by helping identify the cells expressing such signatures in single‐cell expression data.

The data collected in this work represents a state‐of‐the‐art in transcriptomic studies related to prognosis and is also useful for researchers wishing to undertake studies to validate certain transcriptomic signatures in larger or more specific patient samples. However, among the articles we analysed, no studies explored the correlations between specific alternative splicing isoforms of a gene and prognosis in endometrial carcinoma patients and few studies focused on copy number variations (CNVs) to explain alterations found in gene expression. Although extracellular vesicles are involved in various tumour‐related processes, we did not find genes that indicate their involvement, such as CXCR4, EPCAM, TSPAN8, Tsg101, CD9, CD63, CD81, Rab27A [51, 52, 53]. Among the studies evaluating gene expression from biopsy tissue, many used formalin‐fixed and paraffin‐embedded (FFPE) samples, a method commonly used but that may lead to the degradation or fragmentation of RNA, which can significantly impact the quality and quantity of the extracted material [54]. As a result, this variability can introduce sources of error or inconsistency in subsequent gene expression analyses, potentially affecting the reliability and reproducibility of the findings.

The integration of advanced technologies, such as single‐cell RNA sequencing (scRNA‐seq), could provide new insights allowing to attribute a functional meaning to cell groups. Therefore, this database can be useful for tools that perform signature searching and scoring on samples from scRNA‐seq data. In particular, tools such as AUCell, UCELL, and JASMINE [18, 55, 56] have been developed to address this need by providing quantitative scores that measure the activity of predefined gene signatures in each cell. In other words, these scoring approaches evaluate whether a given signature, often associated with specific biological pathways, cell types, or functional states, is coordinately expressed at high levels in the same cell, suggesting that the cell may be carrying out a specific biological function. The application of these scoring methods is critical for dissecting cellular heterogeneity in complex tissues, such as tumours, that are composed of diverse cellular subpopulations exhibiting distinct molecular and phenotypic features, for example, variable proliferative capacity, metastatic potential, immune evasion, and drug resistance. The assignment of scores enables a more refined classification of subpopulations of tumour cells, uncovering the underlying biological mechanisms driving tumour progression and therapeutic resistance. This approach offers also several clinical advantages. For example, in a patient diagnosed with endometrial cancer, single‐cell profiling of the tumour tissue followed by scoring using prognostic signatures from SCENE database can reveal the presence of aggressive tumour subclones expressing gene signatures associated with poor prognosis. This granular information can refine risk stratification beyond traditional bulk tissue analysis, which averages signals across heterogeneous cell populations. Consequently, clinicians can make more informed decisions regarding the aggressiveness of the treatment plan, such as the need for adjuvant therapies or closer surveillance for high‐risk patients. In addition, SCENE and the scoring tools enable longitudinal tracking of prognostic signature activity during treatment. If resistant or aggressive subclones emerge or expand, their identification at the single‐cell level can prompt timely modifications in therapeutic strategy, potentially improving patient outcomes. Moreover, as our database also contains differential expression data between healthy subjects and patients, it could be used for tumour cell recognition. One alternative method for identifying tumour cells is experimental CNVs identification, although when expression data is available only their inference is possible [57, 58].

This research proposes a database specifically designed for endometrial cancer, which allows us to assay the phenotype of each single cancer cell and predict or understand its behaviour, and highlights the importance of transcriptomic profiles in improving the prognosis of patients with EC. In order to ensure the continuous development and relevance of the SCENE database, we are committed to regularly updating it with new transcriptomic signatures associated with EC prognosis. In addition, we welcome user contributions in the form of article suggestions, which will be carefully evaluated by us and will help to keep the SCENE database up‐to‐date, complete, and of high quality.

## Author Contributions

**Erica Dugo:** data curation (equal), formal analysis (equal), investigation (equal), methodology (equal), writing – original draft (equal). **Francesco Piva:** conceptualization (equal), supervision (equal), writing – review and editing (equal). **Matteo Giulietti:** data curation (equal), methodology (equal), software (equal), writing – review and editing (equal). **Luca Giannella:** data curation (equal), methodology (equal). **Andrea Ciavattini:** conceptualization (equal), supervision (equal), writing – review and editing (equal).

## Ethics Statement

The authors have nothing to report.

## Consent

The authors have nothing to report.

## Conflicts of Interest

The authors declare no conflicts of interest.

## Data Availability

The data presented in this study is comprehensively shown in this article and on the website http://www.introni.it/EC/SCENE.html.
